# An American Sensation

**Published:** 1892-03-12

**Authors:** 


### The Hospital

A Weekly Journal of

Science, Medicine, Nursing, and Philanthropy.

*For Hospitals, Asylums, Infirmaries, Dispensaries, Medical Practitioners, Students, Nurses, and the Charitable Public.*

Vol. XI.—No. 285.] [March 12, 1892.

### An American Sensation.

Certain members of the medical profession in America appear to be determined that nobody, not even criminals, shall pass out of the world without their supervision, and, if need be, their active help. The report that comes to England of one of the latest executions by electricity in New York is not calculated to raise our opinion either of the humanity, the common sense, or the scientific capacity of those American physiologists. A criminal is no doubt generally a person deserving of little consideration, and a criminal whose deserts are such as to demand the services of an executioner must be one of the worst of his class. But, however it may be with super-scientific persons, the man who is endowed with the ordinary attributes of human nature feels that even a criminal, when he has been hopelessly condemned to death, has already to a large extent paid the penalty of his crimes, and may not unnaturally be the object of a little sympathy both before and during the act of his execution.

Mercilessness, the absence of the feeling of pity, is one of the most unamiable and dangerous developments of an over-scientific class. If the reports in the newspapers are strictly accurate we have to picture to ourselves a man condemned to die by electricity, sitting pinioned and strapped down in the presence of prison officials, newspaper reporters, and one or more physicians. The man is awaiting, with such feelings as we may imagine, the inevitable stroke of doom. It is at a moment like this that a medical electrician steps forward, and without one thought for the tragic state of mind of the prisoner, proceeds to point out what he appears to consider the "scientific" aspects of the case. "We purpose to day," says he, "to vary the method employed here by making the contact through the hands instead of from the top of the head to the leg as has been the custom before." How much longer this "scientific lecturer" continued his exposition we are not informed, but it was so long that the victim could bear it no longer. "Let her go," he screamed in agonised tones, and then even the "scientist" was moved. The order was given to turn on the electric current, and such was its effect that the unfortunate wretch "bounded up in frightful convulsions, all his muscles straining to break his bonds, his chest arching until the strap that held it threatened to give way under the tension." His face was contorted with an agony impossible to describe. At the end of fifty seconds the current was shut off. Then the chest slowly collapsed, and gasping sounds issued from the throat. "Turn it on the other way," said the physician-executioner. The current was turned on the other way, and the frightful convulsions were repeated once more. "I think," said the doctor, "every physician here will agree with me that the subject was dead so far as conscious being was concerned when the first shock reached him." After the second shock he was "dead muscularly also, that is, as dead as an animal."

The Westminster Assembly of Divines defined the Deity as a being "without body, parts, or passions." The electrical scientist, as he is produced in America, seems to be ambitious to merit at least a portion of this peculiar definition. It is manifest that he prides himself upon being superior to "passions," or at least, to some passions. It is one of the characteristics of a properly cultured physician that he can look upon blood without a qualm, upon the sight of suffering with equanimity, and even upon death itself with comparative indifference. That is entirely as it should be; and this peculiar attribute of the physician has nothing very wonderful about it; it is part of our trade; it is entirely a result of familiarity and routine. So far from there being any peculiar merit in it, one often finds that the grossest, the stupidest, the least intellectual, and the least noble members of the medical profession are endowed with the attribute in the highest degree. It is probable that the American physician who gave his expository lecture on the victim the very moment before execution, prided himself upon being entirely superior to all the emotional states of mind which would have affected an ordinary person called upon to perform a duty under such trying circumstances. And if the physician had but carried out his painful function calmly and silently, without obtruding his miserable lecture upon the consciousness of the dying man, one would have felt that his calmness was a worthy calmness, and in every way befitting the occasion. Even if he had lectured to the living, when the ears of the dead had ceased for ever to be conscious of the sound of his voice one might have held him excusable, though hardly of the finest sensibility in his choice of a moment for unimportant speech. But for him to stand in the presence of his living and sentient fellow-man, a man ignorant of science and philosophy alike, and paralysed with abject terror by the mere contemplation of his impending doom—to stand and deliberately lecture on the mode of death at such a time was senseless, inhuman, a disgrace to science, and worthy of all men's reprobation and contempt.

The student of history is familiar with the often-repeated experience that men are apt to be carried beyond their depth by the prevailing tides of their particular epoch. The strongest current of our own day is that which sets in the direction of unemotional science. We are all becoming insistently critical. Even the poet and the preacher are tempted to cut off their wings and to try the safer method of walking on solid ground. Naturally the man of science is in the ascendant; and it need surprise no one if he sometimes leaves the solid ground and, unmindful of his physical and emotional attributes, essays to live in the passionless atmosphere of cold and abstract reason. But it cannot be done; and thankful we may be that it cannot. Men and women are destined always to feel as well as to think and reason. Moreover, we are all indissolubly bound together by the common ties of a physical organism, a reasoning capacity, and a moral nature. The very best of us cannot sever himself from these impedimenta, and rise above them; nor can the very worst cut himself off in an opposite direction, and sink below them. No man can be entirely independent of the sympathy of his fellow men by reason of his greatness; and no man can be so utterly destitute of all human attributes as to be unworthy of some small grains of sympathy by reason of his baseness. Humanity, as we have so often contended here, is, and for ever must be, one and indivisible.

In past times religion played the part of the teacher and leader of mankind. Science now essays to take this double task upon herself. But are we to be called upon to face the possibility of less worthy teaching, less worthy leading, and a less worthy humanity at the hands of science than religion has shown to the world? This is what seems to be our prospect if men of science are to tear up all human sympathy by the roots, and to lead us into an arid region where all emotion of whatever kind is to be held as a mark of the uninitiated. Practical men judge by practical results, and the world, of which we form the reasoning and ruling element, is first, and above all things, practical. Men hate the abstract when it cannot be reduced to the concrete without injury and misery to living creatures. Many systems and many leading ideas have prevailed for a time, and had a brief day of authority and power. But not one has ever been known permanently to prevail which has trampled upon any single essential attribute of humanity. Science herself, though she looks so fair and promises so much, may yet, like an outworn creed, be ground under the hoofs of the ignorant and the vulgar if she cannot make herself serviceable to the common cause. Men without humanity, whatever their knowledge, are and always must be, hateful.

England has not adopted from America the experiment of execution by electricity. The methods of execution in use in civilised countries, such as decapitation, hanging, poisoning, and the like, satisfy the most important humane conditions of a stern necessity. Compared with execution by electricity, hanging seems to result in a painless and immediate death. The agonised contortions of the latest victim of the New York scientists were believed by one of the doctors present to have been preceded by a complete loss of consciousness. But what experiment can ever assure us that the loss of consciousness on the first impact of the shock is immediate and complete? If it be possible that consciousness continues for fifteen seconds, the indescribable agony of the victim must make even a period so short as that feel like a limitless eternity. But of what avail is that agony? Of what conceivable use is it to either the practical or the scientific world? We are not, however, objecting to execution by electricity if humane spectators are satisfied that it produces painless and speedy death. What we do object to, and what every sober and intelligent human being must object to, is the exquisite cruelty of a scientific expert who can explain to an audience at the moment of doom the scientific peculiarities of a method of execution in the hearing of the victim who is to suffer death.

